# The hierarchical sparse selection model of visual crowding

**DOI:** 10.3389/fnint.2014.00073

**Published:** 2014-09-25

**Authors:** Wesley Chaney, Jason Fischer, David Whitney

**Affiliations:** ^1^Vision Science Graduate Group, University of California, BerkeleyBerkeley, CA, USA; ^2^Department of Brain and Cognitive Sciences and McGovern Institute for Brain Research, Massachusetts Institute of TechnologyCambridge, MA, USA; ^3^Department of Psychology, University of California, BerkeleyBerkeley, CA, USA

**Keywords:** attention, visual attention, coarse coding, ensemble coding, summary statistics, perception, neural network

## Abstract

Because the environment is cluttered, objects rarely appear in isolation. The visual system must therefore attentionally select behaviorally relevant objects from among many irrelevant ones. A limit on our ability to select individual objects is revealed by the phenomenon of visual crowding: an object seen in the periphery, easily recognized in isolation, can become impossible to identify when surrounded by other, similar objects. The neural basis of crowding is hotly debated: while prevailing theories hold that crowded information is irrecoverable – destroyed due to over-integration in early stage visual processing – recent evidence demonstrates otherwise. Crowding can occur between high-level, configural object representations, and crowded objects can contribute with high precision to judgments about the “gist” of a group of objects, even when they are individually unrecognizable. While existing models can account for the basic diagnostic criteria of crowding (e.g., specific critical spacing, spatial anisotropies, and temporal tuning), no present model explains how crowding can operate simultaneously at multiple levels in the visual processing hierarchy, including at the level of whole objects. Here, we present a new model of visual crowding—the hierarchical sparse selection (HSS) model, which accounts for object-level crowding, as well as a number of puzzling findings in the recent literature. Counter to existing theories, we posit that crowding occurs not due to degraded visual representations in the brain, but due to impoverished sampling of visual representations for the sake of perception. The HSS model unifies findings from a disparate array of visual crowding studies and makes testable predictions about how information in crowded scenes can be accessed.

## INTRODUCTION

**FIGURE 1:**
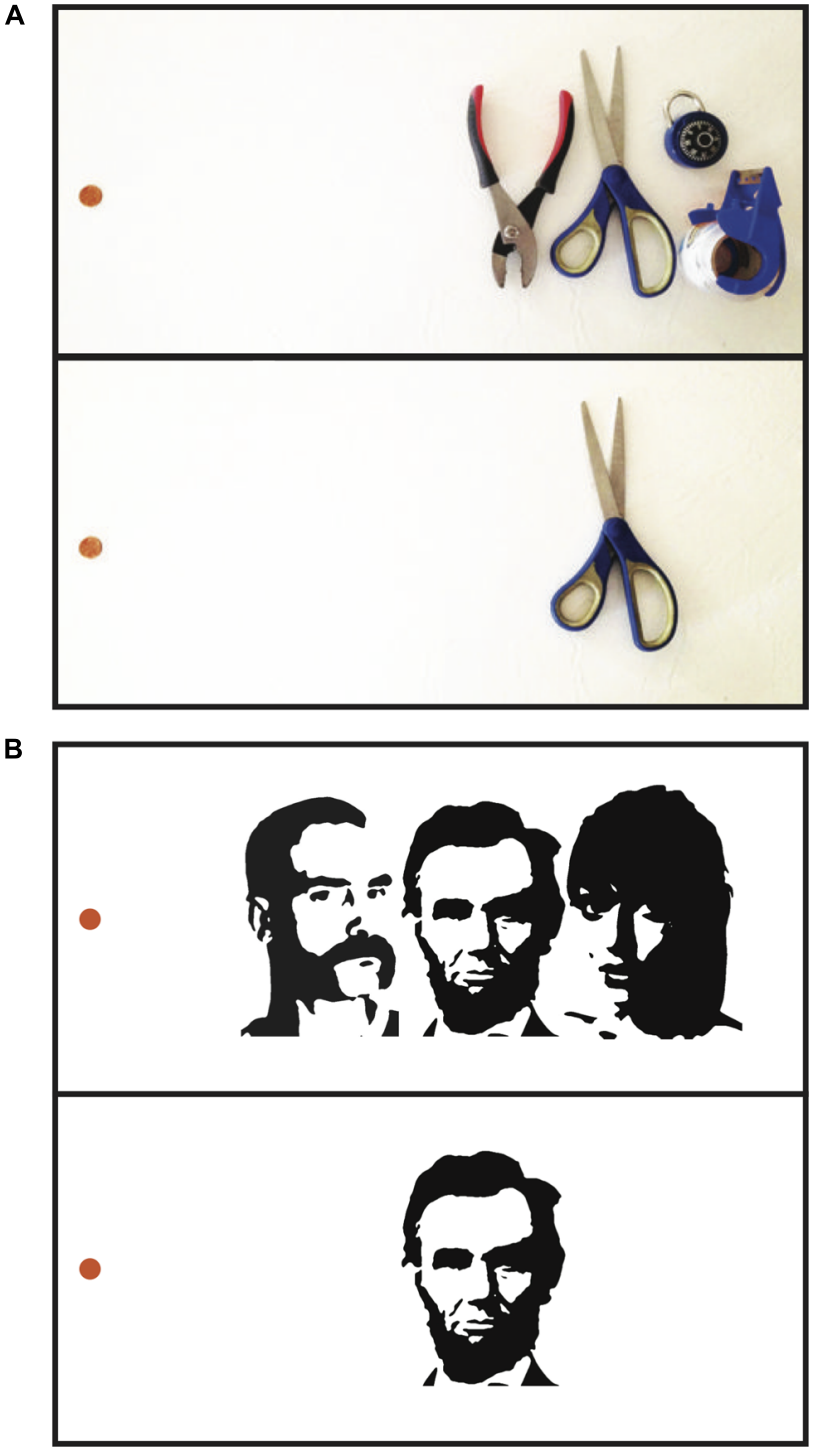
**Visual crowding. (A)** When fixating the penny on the left in the upper panel, the center object on the right is difficult to identify, although it is clear that something is present in the center. In the lower panel, in the absence of surrounding clutter, identifying the same object at the same eccentricity is much easier. Crowding impairs the ability to recognize (but not the ability to detect) objects amidst visual clutter. **(B)** Faces crowd each other. While fixating the orange dot in the upper panel, it is relatively difficult to recognize the identity of the central face due to crowding from the flanking faces. In the lower panel, in the absence of flanking faces, the central face is easier to identify. Crowding between faces is not simply due to crowding of low-level features such as edge information – inverting or scrambling the flanking faces, which preserves low-level features but disrupts holistic face information, reduces crowding between faces (Louie et al., 2007; Farzin et al., 2009; Fischer and Whitney, 2011).

Peripheral vision is not what it seems. Despite the subjective experience of seeing rich detail throughout the visual field, if we are pressed to report the identity of one individual object among others in the periphery, we are very often unable to do so due to the phenomenon of crowding (Levi, 2008; **Figure 1**). Crowding occurs when an object appears among clutter; we lose individual access to the identities of objects spaced too closely together. Access to individual objects is replaced with access to textures of objects – we have an impression of the kind of “stuff” that occupies different regions of space, but no awareness of individual items (Cavanagh, 2001; Tyler and Likova, 2007; Balas et al., 2009; Greenwood et al., 2009; Freeman and Simoncelli, 2011). Crowding imposes a fundamental limitation on our ability to identify objects in everyday life (Whitney and Levi, 2011).

Yet there is another sense in which our visual experience in the periphery is misleading: the experience of crowding seems to imply that the brain simply lacks the bandwidth to represent individual objects outside of those that we scrutinize at the fovea; indeed, nearly all current models of crowding posit that the experience of crowding reflects an underlying irreversible loss of information due to a visual processing bottleneck (He et al., 1996; Levi, 2008; Pelli, 2008; Balas et al., 2009; Greenwood et al., 2009; Freeman and Simoncelli, 2011; Nandy and Tjan, 2012). However, emerging research shows that much more information survives in the periphery than previously thought, albeit sometimes outside the reach of conscious awareness. One clue comes from the fact that we can readily recognize objects that require configural processing, such as faces, when we see them in the periphery (McKone, 2004; Louie et al., 2007), despite the fact that the features of a face in the periphery crowd each other (Martelli et al., 2005). How does the brain reconstruct the arrangement of the features of a face after those features have been jumbled together? That crowding happens at many different representational scales, occurring between basic features (Levi, 2008), object parts (Martelli et al., 2005), and whole objects (Louie et al., 2007; Farzin et al., 2009; Wallace and Tjan, 2011), is paradoxical if crowding at early stages of visual processing destroys the information required by higher-level stages.

We recently directly tested the degree to which object-level information can survive crowding for use in subsequent visual processing (Fischer and Whitney, 2011). We presented sets of faces in the periphery and asked observers to report either the expression of an individual (crowded) face from the set, or the average expression of the set as a whole. We found that even for sets of faces where observers were at chance in discriminating the expression of the crowded face that particular face contributed with high precision to the perceived average of the set, an effect that cannot be explained by a contribution of low-level features alone. Individual object information is not lost amid the clutter in the crowded periphery, it is simply inaccessible to perception. In support of these findings, another recent study found that illusory contour formation, a process that relies on the configuration of the inducer stimuli, can also survive crowding of the individual inducers (Lau and Cheung, 2012) [but see (Banno and Saiki, 2012) for data suggesting that size information does not survive crowding]. Further, crowded objects can unconsciously influence behavior by priming subsequent responses (Faivre and Kouider, 2011; Yeh et al., 2012) and biasing preferences (Kouider et al., 2011).

Thus, a satisfactory theory of crowding must account for not only for the perceptual degradation that crowding produces, but also for how certain information survives crowding and can contribute to downstream processes. The most prominent current models of crowding posit that crowding results from excessive integration of information appearing in the periphery, due to the number of neurons representing a given location in space (Pelli, 2008; Pelli and Tillman, 2008), lateral connections shaped by image statistics during development (Nandy and Tjan, 2012), or the resolution of visual attention (He et al., 1996). Some over-integration models can successfully account for most or all of the classical properties of crowding, but all posit information loss due to a resolution bottleneck, and thus cannot explain how crowded visual features or objects can be available with high fidelity to downstream processes. Another related model of crowding, the positional averaging model (Greenwood et al., 2009), posits that crowding results from pooling position information to reduce positional uncertainty. Positional averaging may also account for object-level crowding (Dakin et al., 2010), but it still posits information loss, and cannot account for how holistic object information survives crowding and influences ensemble perception (Fischer and Whitney, 2011). Thus, while the general idea of involuntary pooling captures many aspects of crowding and likely plays a role, over integration is not the whole story. Other models of crowding, including substitution (Wolford, 1975; Chastain, 1982) and contrast-gain or masking based models (Krumhansl and Thomas, 1977; Chastain, 1981; Petrov and Popple, 2007) are not more successful; they similarly require that information about crowded objects is lost or substantively modified, a prediction that has been overturned (Faivre and Kouider, 2011; Fischer and Whitney, 2011; Kouider et al., 2011; Lau and Cheung, 2012; Yeh et al., 2012).

Here we propose a new model of visual crowding, the *hierarchical sparse selection (HSS) model*, in which unconscious object processing continues unencumbered by clutter in the scene. Our model accounts for the known characteristics of crowding, and generates several predictions for future tests (**Box 1**).

**Box 1. HSS model predictions.**

The HSS model makes a number of concrete predictions at both the behavioral and neural levels for future testing:

(1) The HSS model predicts that crowded stimuli are represented robustly in the brain even though they are blocked from conscious individuation. Thus, it should be possible with both fMRI pattern analysis and neurophysiological recordings to find precise representations of crowded objects in the brain.
(2) The HSS model predicts that the critical spacing of crowding is different for different stimulus categories (e.g., gratings, faces, bodies, objects, etc.) because crowding is a function of receptive field size within the cortical map in which the stimulus is represented. There is already some evidence that critical spacing differs across stimulus categories [see (Whitney and Levi, 2011) for a review], but the spatial extent of crowding has not yet been precisely characterized for a wide variety of stimuli, nor has there been a test of the relationship between receptive field size and the extent of crowding across stimulus categories.
(3) In the HSS model, precise information about crowded objects persists in the visual processing stream despite the perceptual experience of crowding. Thus, information about crowded targets may be available to other processes in addition to ensemble perception and priming. For example, action may not suffer from crowding as much as perception (Bulakowski et al., 2009).
(4) A prediction of the HSS model is that with extensive experience viewing a particular stimulus category at a particular position in the visual field, it may be possible to reduce crowding through training. If information about a crowded target is present but requires fine-tuned connections to decode, it may be possible to train up the required connections. However, such training should not transfer to other sufficiently different stimulus categories even at the same spatial location because crowding depends on connections to the particular map that the stimuli are represented in. There is indeed evidence that training can reduce the strength and extent of crowding (Wolford et al., 1988; Chung, 2007; Hussain et al., 2012), but the specificity of the reduced crowding to object category remains to be tested.

## THE HIERARCHICAL SPARSE SELECTION MODEL OF VISUAL CROWDING

**FIGURE 2:**
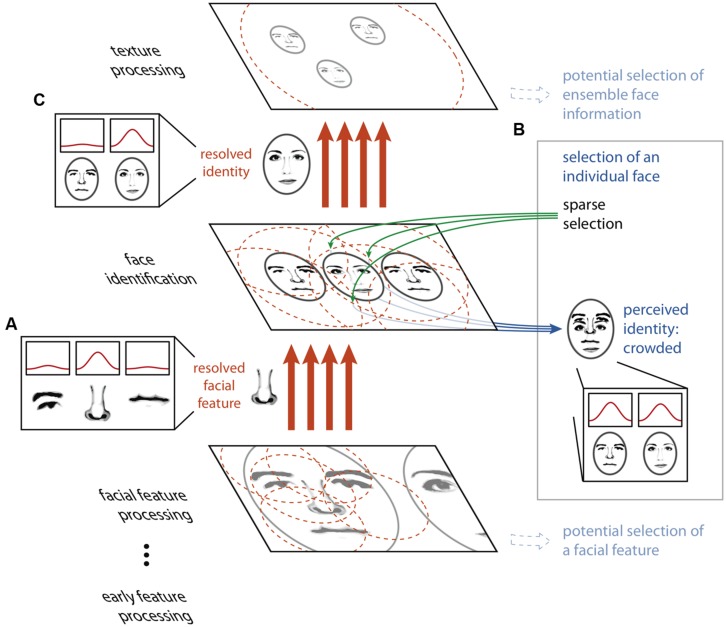
**The HSS model of visual crowding.** Unambiguous information about features or objects is passed between processing stages via an ensemble- or coarse-coding scheme, using a sufficient number of receptive fields with dense connections to avoid information loss through over-integration **(A)**. Perceptually accessing an object from a given map relies on a sparse selection of a subset of the receptive fields or connections from that particular map only, resulting in the read-out of an ambiguous conscious percept **(B)**. Thus, an object that is perceptually crowded can nonetheless be passed, intact, to a subsequent texture processing stage **(C)**.

Our proposed model rests on two principles. First, large receptive fields or integration regions do not imply the loss of fine-scaled information. While it is true that the output of a single neuron with a large receptive field will carry highly integrated, spatially, and featurally ambiguous information in the presence of visual clutter, a population of many such neurons can carry sufficient information to resolve details on a scale far smaller than the receptive field size. Indeed, the feature or object at a precise location can be isolated from amongst clutter by combining the outputs of many highly overlapping receptive fields, as has been described in detail in the ensemble- and coarse-coding literature (Eurich and Schwegler, 1997; Pouget et al., 2000; Purushothaman and Bradley, 2004), and large receptive fields may in fact be a more efficient means of carrying fine spatial information than small receptive fields (Baldi and Heiligenberg, 1988; Snippe and Koenderink, 1992; Eurich and Schwegler, 1997). **Figure 2A** depicts this concept: neurons tuned to facial features have receptive fields that cover many features at once for a face seen in the periphery. Each individual neuron signals ambiguous information about the features present at a given location, yet with a proper decoding scheme, a combination of the outputs of many neurons can resolve the feature present at a given location. Thus, object processing can proceed unencumbered by clutter given precise enough wiring from one stage to the next. This notion is consistent with the fact that higher-level visual areas that are closely tied to the perception of object identity and position (Williams et al., 2007; Fischer et al., 2011; Maus et al., 2013) have large receptive fields even in central vision (Raiguel et al., 1995; Amano et al., 2009), yet we can resolve and identify closely spaced objects in central vision.

**FIGURE 3:**
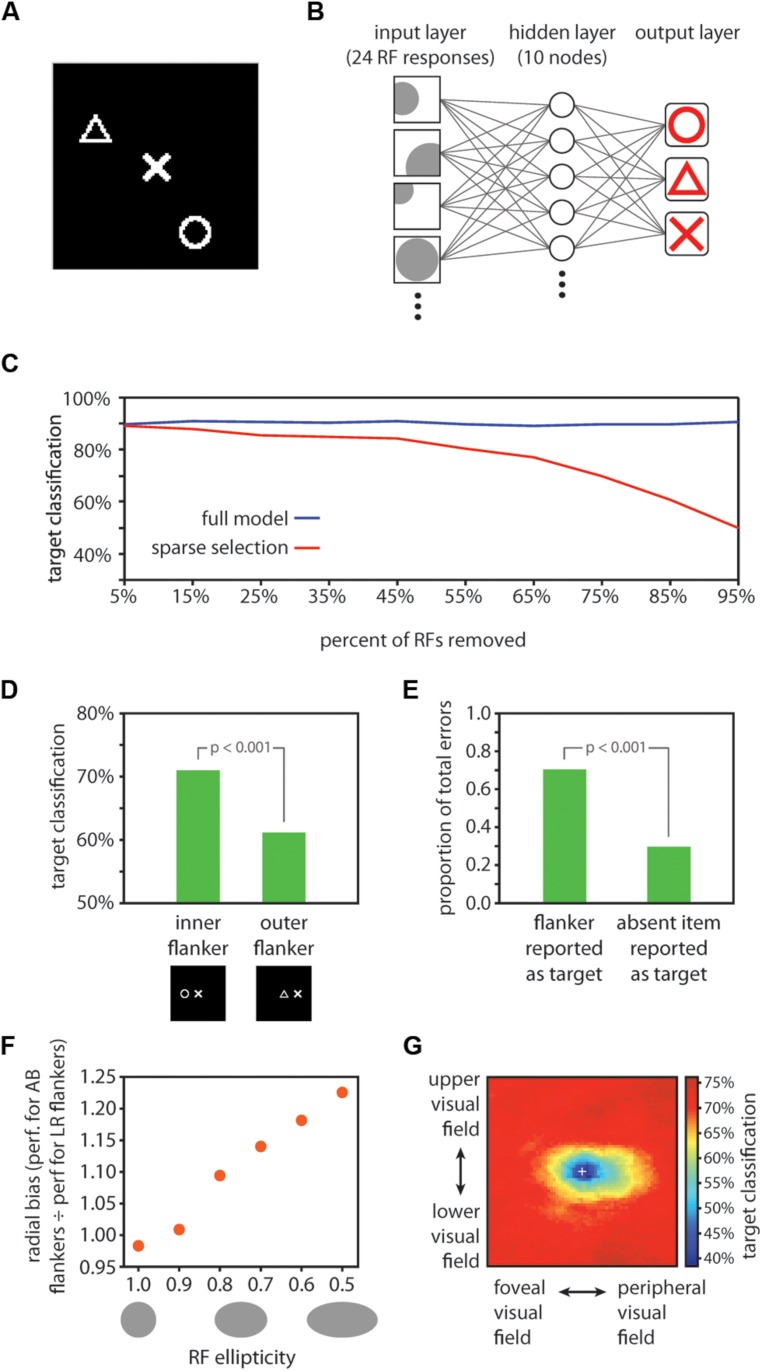
**Computational model results. (A)** Example display for model training and testing. One symbol (the target item) was positioned in the center of the image, and two other randomly selected symbols (flankers) were positioned in random locations elsewhere in the image. **(B)** Schematic depiction of the neural network decoding model. Twenty-four receptive fields were tiled in random locations over the image. RFs had random tuning functions, but an equal number of RFs were optimally tuned to each of the three symbols (8 RFs optimally tuned to each of circles, triangles, and Xs). The model contained a ten unit hidden layer and a three unit output layer. Each node in the hidden layer was connected with each of the 24 RFs, and with each of the three nodes in the output layer. The three output layer nodes corresponded to the three symbols, and stimulus decoding was determined by taking the maximally responsive node in the output layer in a winner-take-all fashion. **(C)** Comparison of performance for the full model vs. the sparse selection model. While the full model classified the target symbol with high accuracy (90.4% correct) despite the presence of flankers (blue data), classification performance of the sparse selection model decreased monotonically as more connections were removed (red data). Thus, although fine-scaled information can be decoded from a population of neurons with large receptive fields, robust decoding of crowded stimuli relies on a full sampling of the information present in the neural population. **(D)** RF scaling with eccentricity yielded the inner-outer asymmetry characteristic of visual crowding. We tested the model performance using images with one flanker positioned either on the foveal side of the target symbol (here shown to the left of the target symbol) or on the eccentric side of the target symbol. Classification was significantly worse when an outer flanker was present vs. an inner flanker (*p* < 0.001), mirroring effects found in human performance (Bouma, 1973; Petrov et al., 2007).** (E)** Using the same test images as those used to test the inner-outer asymmetry, we found that when the model made a classification error, it was significantly more likely to report the flanker as the target than to report the symbol that was absent from the display (*p* < 0.001). This is consistent with human performance; observers frequently substitute a flanker for the target in a crowded display (Wolford, 1975; Chastain, 1982).** (F)** We varied RF ellipticity in the model from 1.0 (circular) to 0.5 (half as tall as wide). For each value of RF ellipticity, we tested model performance with images in which flankers were positioned either to the left and right of the target (LR flankers; positioned along the radial dimension relative to the fovea) or above and below the target (AB flankers; positioned along the tangential dimension). We computed the ratio of performance when AB flankers were present to performance when LR flankers were present as a measure of radial bias in model performance. Radial bias increased monotonically as RFs became more elliptical, demonstrating that asymmetrically shaped RFs are a plausible source of the radial bias in crowding. However, the validity of the HSS model does not hinge on elliptical RFs. Other potential sources of the radial bias in crowding such as saccadic influences on the development of lateral connections (Nandy and Tjan, 2012) could be similarly integrated into the HSS model. **(G)** A visualization of the crowding zone based on the neural network model performance (the region of space around the target within which the presence of a flanker crowds the target). The white cross marks the location of the target; model performance was tested with a single flanker positioned at every possible location within the display. Here, we expanded the size of the display space by 50% relative to previous tests of model performance in order to visualize the full extent of the crowding zone. The visualized crowding zone is reminiscent of the elongated spatial interaction zones found by Toet and Levi (1992).

If high-fidelity information can be transmitted through a neural system with large receptive fields, why does crowding occur? The second component of our proposal is that while the feed-forward cortical object processing hierarchy possesses the copious and fine-tuned connections necessary to resolve the relevant features at every stage, the operation which “reads out” selected cells’ outputs to conscious perception does not. Key to this notion is that within a coarse coding framework, unambiguous features and objects need not be explicitly represented by individual neurons at any stage of processing. Rather, information about an individual visual feature is encoded across a population of cells, and this information is decoded *between* stages of processing by the precise pattern of connections between neurons in one stage and the next. In the example in **Figure 2**, no single neuron at the facial feature processing stage unambiguously represents the nose, nor does any single neuron at the face identification stage. However, the presence of the nose at its precise location is conveyed between the facial feature processing and face identification stages by a specific and finely tuned pattern of connections. If the selection of information from a given map for perception relies on connections to a subset of the units in this map (a “sparse selection”), there may be insufficient information available to unambiguously decode the selected feature (**Figure 3B**). Thus, it is only possible for an observer to perceptually individuate an object when it can be unambiguously decoded from this limited sparse selection of the information in the neural population representing it, and this requires that the object is sufficiently separated from the clutter around it. However, object processing carries on regardless of whether this condition of sufficient separation is met (**Figure 3C**). It is important to differentiate sparse selection from the unrelated notion of sparse coding. Here, by “sparse selection” we mean capitalizing on information from a limited and sometimes insufficient number of units, whereas “sparse coding” refers to a sufficient coding scheme that favors having the smallest number of active units possible.

Why would perceptual selection only sample a subset of the relevant information available for resolving objects in the periphery? There are two likely reasons: First, attention must be highly flexible, able to select any feature from any position in the visual field. The number of connections required to perfectly sample information from any visual map in the brain is prohibitive. Putative attentional regions in the fronto-parietal network (Corbetta et al., 1993; Buschman and Miller, 2007) and the pulvinar (Petersen et al., 1987; Fischer and Whitney, 2012) possess widespread connectivity throughout the brain, but connect with only a subpopulation of the cells in a given brain region (Curcio and Harting, 1978; Schall et al., 1995; Kaas and Lyon, 2007). Second, the integrated ensemble information that we perceive in the periphery is useful for providing a rapid gist of the scene (Oliva, 2005), as well as guiding attention and saccades (Torralba et al., 2006). Trading off individual object information for ensemble representations in the periphery might be a benefit rather than a hindrance.

Importantly, our proposal is not that crowding results from the same limit on the spatial resolution of attention proposed by He et al. (1996). Their model asserts a smallest area of the visual field over which attention can operate; our model is about the sparsity of sampling within that region. Attentional sampling could be highly spatially specific, yet if attention samples from a limited number of receptive fields at the selected location, the object at that location cannot be resolved. Further, the sparse selection we propose can happen at any level of processing and is not limited by a single resolution of attention. It is the size of the receptive fields at a specific level of analysis, coupled with a sparse sampling of the information represented at that level of analysis for perceptual access that causes crowding. The HSS model predicts that the critical spacing for crowding (the maximum distance at which a flanker can be positioned from the target and still cause crowding, as a function of eccentricity) differs for different stimulus classes (see Discussion), whereas the attentional resolution model predicts a single critical spacing for all stimuli based on the smallest possible attentional window at a given eccentricity.

### COMPUTATIONAL MODEL

To test the outcome of drawing a sparse sample from coarse-coded visual information, we constructed a computational model aimed at decoding crowded visual features based on the output of randomly tiled receptive fields.

#### Model construction

The display images were 101 × 101 pixel images consisting of white symbols drawn on a black background (**Figure 3A**). There were three possible symbols: a triangle, an X, and a circle, each drawn within a 20 × 20 pixel area in the images. In all display images, one symbol was placed at the center of the image; this center symbol was the crowded item that the model aimed to decode. In training images, two additional random symbols (flankers) were placed at random locations within the image; the training set comprised 120 such images – 40 images with a triangle at the center, 40 images with an X at the center, and 40 images with a circle at the center. Model testing was conducted on an independent set of 60 images constructed in the same fashion for basic model testing or with the flankers placed at specific locations for testing of asymmetries and substitution errors (described below).

The model consisted of receptive fields tiled over the image space (the input layer) whose outputs were fed into a neural network with one 10 unit hidden layer and a 3 unit output layer (**Figure 3B**). On each iteration of model training and testing, we tiled 24 receptive fields over the image space in random locations. Receptive fields had a mean diameter of 50 pixels. The left side of the image was treated as being more foveal and the right side of the image more eccentric, such that the image represented a patch of the right visual field. Receptive field size scaled linearly with eccentricity with a slope of 0.7, consistent with the scaling in extrastriate object-selective cortical regions (Amano et al., 2009). Each receptive field was preferentially tuned to one of the three symbols but responded to some degree to each of the symbols. The response of a receptive field was computed by convolving a filter (a 20 × 20 image of the symbol that the RF was maximally tuned to) over the entire image and then taking the maximum of the convolution output within the region of the display image that the RF covered. Thus, when the optimal stimulus was present anywhere within an RF, the RF response was 1.0; if the preferred stimulus was partially within the receptive field or a non-preferred stimulus fell within the receptive field, the response was less than 1 but greater than 0. We applied a rectification that mapped negative convolution values (possible if two stimuli fell close together within the RF) to 0. If no stimulus fell within a receptive field, its response was 0.

The set of 24 receptive fields comprised the input layer to the neural network; each RF had a connection to each of 10 units in the hidden layer, and each unit in the hidden layer had a connection to each of 3 units in the output layer (**Figure 3B**). The three output layer units corresponded to the three stimulus categories; stimulus decoding was determined in a winner-take-all fashion on the three output units. Training of the model weights was conducted with scaled conjugate gradient backpropagation implemented with the Matlab Neural Network Toolbox (MathWorks, Natick, MA, USA). Model performance was then taken as the proportion of 60 independent test images correctly classified by the model. We conducted 1,000 iterations of model training and testing, randomizing the stimuli, RF locations, and RF tuning on each iteration, and we report the average model performance across all iterations. To test the significance of the model performance, we generated an empirical chance distribution by shuffling the stimulus labels prior to model training, then testing on an independent set of images with the correct labels. Repeating this shuffling procedure 1000 times produced a distribution of performance estimates that would be expected by chance; the significance of the model performance was taken as the proportion of the chance distribution that was larger than the actual estimated model performance.

To test whether the same model predicts crowding at the fovea, we adjusted the overall display size to 61 × 61 pixels from 101 × 101 pixels to keep target, flankers, and receptive fields within a smaller eccentricity range and closer to the fovea. The same three images were used (white circle, X, and triangle on black background) at the same sizes as before (20 × 20 pixels each). The target was presented in the center of the display image with two flankers randomly placed in non-overlapping positions. The number of receptive fields in the model was increased from 24 to 45. This increase combined with the reduction in overall display image size lead to an increase in RF density (ratio of number of RFs to pixel area) by a factor of 5, consistent with an estimate of cortical magnification from V1 (Sereno et al., 1995; Engel et al., 1997; Qiu et al., 2006) assuming target eccentricity of 5° in the previous model and 1° or less in the foveal model. This is a conservative estimate because cortical magnification is greater in extrastriate visual cortex than in V1 (Harvey and Dumoulin, 2011), and object crowding likely occurs beyond V1 (Farzin et al., 2009; Whitney and Levi, 2011). The remainder of the model was left unchanged: we used 10 hidden units, 120 training images, and 60 test images to run 1000 iterations of the model.

Finally, in order to further illustrate and clarify the hierarchical nature of the model, we present simulations of performance on two additional tasks, identifying either features or an object constructed from those features, using the same feature-tuned receptive fields in the input layer. In these simulations, there were two possible tunings for receptive fields, a horizontal line and a vertical line. The display images were again 101 × 101 pixel images with a target at the center. For the feature task, the target and flankers were either a horizontal or vertical lines. For the object task, the receptive field tuning remained the same, but the target and flankers consisted of “tumbling Ts”: the letter T oriented in one of the four cardinal directions. The size of the receptive fields was reduced to an average of 20 pixels diameter and the number of receptive fields was increased to 48, modeling a region with selectivity for lower-level features. All other aspects of the model were identical to the original implementation and we tested the model by performing 1000 iterations with randomized target and flanker identities, flanker locations, and receptive field locations within the 101 × 101 display image.

#### Model performance

Target shape decoding performance was 90.4% correct, significantly greater than chance (chance performance = 33.3% correct; *p* < 0.001). This result establishes that target identity in a cluttered array can be resolved from the pooled output of a population of RFs, even when no individual RF is small enough to encompass the target alone. To test the effect of sparse sampling from the simulated neural population, we repeated the above analysis, this time removing a portion of the receptive fields from the network and then retraining (assigning new connection weights) after the removal of units and prior to testing. This procedure simulates the case where decoding of stimuli for conscious perception relies on a network of connections entirely distinct from that of feed-forward processing, connected to a sparsely selected subset of units. The results of this analysis are shown in **Figure 3C**: reducing the number of units sampled for the readout of the crowded central target led to a monotonic decrease in model performance, with performance dropping to 90% of the full model performance when 85% percent of the input units were sampled. Removing a portion of the receptive fields from the trained network without retraining prior to testing (simulating the case where attentional selection taps into the same network that robustly represents the target identity, but only has access to a subset of the units in the network) produced a comparable pattern of results. Similarly, removing individual connections rather than entire RF units from the model also resulted in a monotonic decrease in performance, though at a slower rate than removing entire receptive fields. The principle of “sparse selection” therefore holds irrespective of whether it is entire units or individual connections between units that are selected. In short, decoding target identity from a population of cells requires connections with a sufficient proportion of the cells to resolve those stimuli that are spaced closer together than the size of a receptive field.

We next asked if model performance followed the well-established property of inner-outer asymmetry: a flanker presented in a more eccentric location relative to the target produces stronger crowding than a flanker presented at the same distance from the target but in a more foveal position (Bouma, 1973; Petrov et al., 2007). To test for an inner–outer asymmetry, we trained the model in the same fashion as above, but tested on images with just one flanker, positioned either 25 pixels to the left or 25 pixels to the right of the target. In this case the flanker was not allowed to be the same symbol as the target; thus, there were 12 total images in the test set. The sparse selection model for this and subsequent tests was generated by dropping a random selection of 50% of the RFs in the full model post-training. A comparison of model performance for test images where the flanker was more foveal than the target (positioned to the left) vs. the images where the flanker was more eccentric revealed an asymmetry in line with psychophysical results: the presence of an eccentric flanker yielded significantly worse model performance (*p* < 0.001; **Figure 3D**). This asymmetry was absent without sparse selection – the inner/outer asymmetry emerges from the model as a result of the interaction between receptive field eccentricity scaling and sparse selection.

Another well-established aspect of crowding is that when observers make errors in reporting a crowded target, they report a flanker rather than another potential symbol with above-chance frequency [substitution errors; (Wolford, 1975; Chastain, 1982)]. Using the same set of test images as described above for testing the inner–outer asymmetry, we asked whether the model more commonly reported the flanker, rather than the third symbol which was not present in the display, when it made an error. This was in fact the case: 70.4% of errors arose from reporting the flanker as the target, rather than reporting the symbol that was not present (**Figure 3E**).

In behavioral tests, flankers positioned radially in relation to the target (e.g., to the left and right of the target for a target appearing on the horizontal meridian) crowd more strongly than flankers positioned tangentially (above and below the target in the same example), an effect known as a radial bias (Toet and Levi, 1992). A simple addition to our model could account for the radial bias in crowding: if receptive fields are elliptical rather than circular (Motter, 2009), elongated in the radial direction, a radial bias emerges in the model performance. We tested this effect by using test images with two flankers either 25 pixels to the left and right of the target or 25 pixels above and below the target. We then varied the ellipticity of the receptive fields in the model from 0 (perfectly circular) to ½ (half as large in the vertical direction as in the horizontal direction). The relative performance for test images with left/right flankers vs. images with up/down flankers decreased monotonically with increasing RF ellipticity. That is, the radial bias in model performance increased with more elliptical RFs, and was significant (a significant departure (*p* < 0.05) from a left/right vs. upper/lower performance ratio of 1, which reflects no bias) with ellipticity values of 0.8 or smaller (**Figure 3F**).

There is strong evidence for elliptical receptive fields throughout the visual processing stream in mammals, for example in V4 of rhesus monkeys (Motter, 2009), in macaque ventral visual areas (Op De Beeck and Vogels, 2000; Pigarev et al., 2002), in areas 7, 21a, and claustrum of cats (Sherk and LeVay, 1981; Rodionova et al., 2004) and in RF subregions in mouse visual cortex (Smith and Häusser, 2010). As such, it is important to incorporate elliptical receptive fields in a computational model of crowding in ventral cortical regions. Ellipticity is one possible explanation for the radial bias in crowding, and it would dovetail with the aforementioned neurophysiological literature. However, there are other potential contributors to the radial bias in crowding such as saccadic influences on the development of lateral connections (Nandy and Tjan, 2012) that could be similarly integrated into the HSS model. Even without elliptical receptive fields, cortical magnification factor in the random placement of the RFs and eccentricity-dependent size scaling introduced some radial bias into our model. Our model does not hinge on any particular mechanism for the production of a radial bias; rather, the HSS model can be thought of as a module that can be added to many current models of crowding in order to extend them to account for how high fidelity information can survive crowding.

Next, we generated a visualization of the spatial extent of crowding produced by the HSS model (**Figure 3G**). We used training and test images that were 150% of the size used in previous model testing (now 151 × 151 pixels); symbols were still 20 × 20 pixels. To accommodate the larger display image space, we increased the number of RFs in the model to 48, and the number of training images to 240. The ellipticity of RFs in the model was set to 0.5. On each of 100 iterations, we trained the model using the 240 training images (each had a target at the center of the image and two randomly positioned flankers), and then tested the model performance on a series of test images in which a flanker was positioned at every possible location in the display image. For each possible flanker location, there were six test images corresponding to all pairings of one symbol type as the target and a different symbol type as the flanker. Within a given flanker location, overall model performance was the % of the six test images correctly classified. In **Figure 3G**, the color at a given location in the image corresponds to the model performance when a flanker was positioned at that location and a target was positioned at the center of the image. The performance shown in **Figure 3G** is average performance over 100 iterations. The resulting visualized “crowding zone” is reminiscent of the elongated spatial interaction zones found by Toet and Levi (1992), and additionally shows an inner/outer asymmetry: the region within which a flanker degrades performance extends further into the periphery than toward the fovea.

**FIGURE 4:**
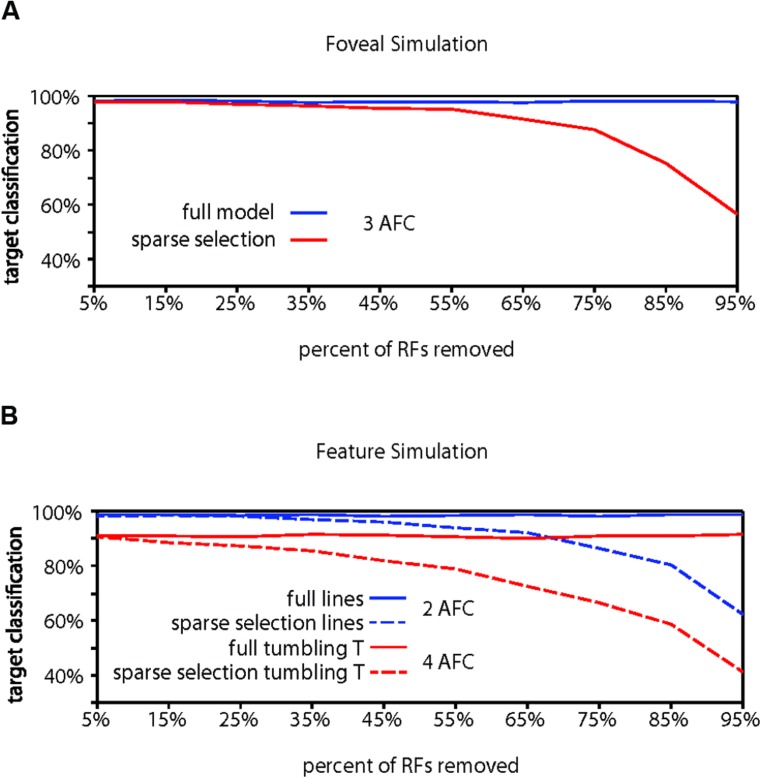
**Computational model results for fovea and feature tuning. (A)** Comparison of performance for the full model vs. the sparse selection model at the fovea. Increased receptive field density yields higher full model performance than in the periphery (98.18%). Sparse selection of receptive field units leads to a much smaller performance decrease demonstrating reduced crowding in the fovea. Increased receptive field density in foveal regions can reduce or eliminate the effect of sparse selection. **(B)** Demonstration that feature-tuned receptive fields can perform well on both a feature discrimination task (98.73%) and an object discrimination task (91.06%) in the presence of flankers (solid lines). Sparse selection of feature-tuned receptive fields leads to a monotonic decrease in performance and crowding of features (dashed blue), showing that the model predicts crowding at any behaviorally relevant level of processing. Sparse selection occurs at only in the perceptual readout from one level and not in the information passed between levels, avoiding the degradation of object level encoding that would occur if object representation depended on the sparse selection of features (shown in dashed red).

Evidence for whether crowding occurs in central vision is mixed (Levi, 2008), but crowding is generally thought to be at least weaker near the fovea than in the periphery. Our foveal model (**Figure 4A**) with a modest increase in RF density and a bias toward locating RFs at lower eccentricities in accordance with the V1 cortical magnification factor (Sereno et al., 1995; Engel et al., 1997; Qiu et al., 2006) showed higher overall target identification performance, correctly identifying a target in 98.18% of trials, significantly greater than chance (chance performance = 33.3% correct; *p* < 0.001). Furthermore, performance in the foveal model required removing 75% of the RF units to reach 90% of the full model performance, 20% more than the peripheral model and equivalent to a 44% reduction of the RFs remaining in the peripheral model. We do not, however, want to stress too strongly the specific values we obtain. The parameters used here reflect extrapolations of cortical magnification and receptive field scaling into the most foveal portion of the human visual field, which affect the performance of the model. Rather, the results should be taken to qualitatively show that increased density of receptive fields and reduction of the size of the receptive fields could explain why sparse selection at the fovea would not result in crowding or would cause much weaker crowding than in the periphery.

Finally, to demonstrate the hierarchical aspect of the model, we conducted a simulation of crowding performance using feature-tuned receptive fields, as opposed to objects or letters. In order to show that the model predicts crowding for features as well as objects, we first trained and tested the model with target and flankers that were horizontal and vertical lines (**Figure 4B**). Overall model performance was 98.73%. The model dropped below 90% of full model performance (88.86%) when 75% of the receptive fields were removed before retraining, indicating that crowding would occur in the identification of horizontal and vertical lines, if there were sparse selection of feature-level information, a simple task and only 2AFC as opposed to the 3AFC tasks in previous simulations. This demonstrates the hierarchical aspect of the model: the model can account for crowding of both features and whole objects when it is applied at any behaviorally relevant level.

The HSS model states that attention sparsely selects from the behaviorally relevant level of the visual hierarchy (**Figure 2B**), not that there is a cumulative effect of sparse selection at each level of the hierarchy. To show why, we trained the full model of this same network with feature detector receptive fields to identify “tumbling Ts” at a surprisingly high 91.06% correct performance (**Figure 4B**). This is a 4AFC task where every target and flanker contains both of the possible features that any given receptive field is tuned to and only relative location information is useful for the task. At 75% removal of feature tuned receptive fields, enough to cause crowding of features, “tumbling T” performance dropped to 66.6% correct. This scenario shows what would happen if degraded feature information was passed forward to subsequent visual processing stages – object-level information would be severely degraded. This contradicts many studies that have demonstrated that object level information gets through the bottleneck of crowding (Fischer and Whitney, 2011). That is, if sparse selection occurred cumulatively at each level in the hierarchy (which is not what we are proposing), it would suffer from the same weaknesses as other crowding models: it could not account for the preservation of object information evidenced by object ensembles, priming, and other effects (Faivre and Kouider, 2011; Kouider et al., 2011; Lau and Cheung, 2012; Yeh et al., 2012)

Because the HSS model of crowding posits that sparse selection occurs only at the behaviorally relevant level of representation (selection occurs at the feature level of representation when the task is to identify a crowded feature and at the object level when the task is to identify the object), the object representation is preserved in the full feed-forward hierarchy. Crowding can still occur at either level through a sparse selection of either feature or object level information for conscious awareness.

The model performance demonstrates that, in principle, the HSS model can give rise to the known properties of visual crowding while supporting the transmission of high precision information within the cortical object processing hierarchy. This computational model is not intended to provide quantitative predictions about the number of neurons required in a coarse-coding framework or the number of neurons sampled by attention, but rather to provide a conceptual verification that: (1) fine-scaled information can be decoded from a population of neurons with large receptive fields, (2) a sparse selection from a neural population with large receptive fields results in rapid degradation of target identification and flanker-target confusions in the periphery but not in the fovea, (3) sparse selection at the behaviorally relevant level of processing nonetheless leaves high-fidelity stimulus information intact in the feed-forward visual processing stream, and (4) properties of receptive field scaling (in this case, larger receptive fields in more peripheral locations) can give rise to the asymmetries that are diagnostic of crowding.

## DISCUSSION

The HSS model accounts for both the broad array of previously known characteristics of crowding and for recent findings that information can survive crowding, influencing ensemble perception (Fischer and Whitney, 2011; Lau and Cheung, 2012), priming behavior (Faivre and Kouider, 2011; Yeh et al., 2012), and biasing preferences (Kouider et al., 2011). The computational implementation of the HSS model described above deals with the simple case of decoding target identity from a small, discrete set of stimuli. The computational model itself is not intended to provide an exhaustive account of how sparse selection leads to crowding, but rather to provide a proof of concept that simply reducing the amount of information sampled for perceptual readout at any particular level of analysis gives rise to many of the known characteristics of crowding.

A hallmark of the HSS model is that it posits that crowding occurs between stimuli that are represented in the same cortical maps but not between stimuli that are represented in distinct maps (here, by “map” we mean an organized representation of visual space and/or basis dimensions *within* an object category). This feature of the HSS model accounts for why flankers of a different object category than the target are not effective crowders (Louie et al., 2007; Farzin et al., 2009). Since categorically different objects and features are coded in separate maps in the cortex (Op de Beeck et al., 2008), a target will be isolated in its cortical map and thus recognizable if the surrounding flankers are sufficiently different to be represented in a different cortical region. Likewise, this feature of the HSS model explains how grouping the flankers into an object can break down crowding (Livne and Sagi, 2007; Saarela et al., 2009) by causing the object formed by the distracters to be processed in a different cortical map than the target. Even when the target and flankers are of the same object category (e.g., a Gabor crowded by Gabors or a letter crowded by letters), a large difference between the target and flankers along dimensions such as color, orientation, and spatial frequency, and others can attenuate crowding (Andriessen and Bouma, 1976; Nazir, 1992; Kooi et al., 1994; Chung et al., 2001; Põder, 2007). This could also be the result of compulsory grouping of the target and flankers into separate objects (Kooi et al., 1994), but another possibility exists: when the target and flankers differ markedly along one of these dimensions, even a sparse sample may be sufficient to successfully resolve the target from the flankers because of the large target/flanker signal difference. The fact that visual “pop-out” can alleviate crowding (Põder, 2007) may simply be due to the target and flankers being different enough to resolve from the sparse sample of neural outputs available to conscious perception.

The HSS model also naturally accommodates the finding that a crowded target can produce adaptation and aftereffects despite being perceptually inaccessible (He et al., 1996; Aghdaee, 2005; Whitney, 2005; Harp et al., 2007; Bi et al., 2009): a crowded object fatigues the same population of cells that it would if it was presented in isolation – the perceptual phenomenon of crowding does not interfere with the underlying stimulus representation.

In sum, we present a novel model for visual crowding which posits that crowding occurs at multiple levels throughout the visual processing hierarchy, rather than at a single bottleneck. Counterintuitively, information about crowded objects is represented robustly in the brain, but may be inaccessible to conscious perception due to a sparse selection of information on which perception relies. The model is not intended to replace all existing models of crowding, but it could be a complementary component of any existing model; the HSS model does help account for many puzzling findings in the crowding literature that have otherwise gone unexplained.

## Conflict of Interest Statement

The authors declare that the research was conducted in the absence of any commercial or financial relationships that could be construed as a potential conflict of interest.
